# Telemetry and Accelerometer Tracking of Green Toads in an Urban Habitat: Methodological Notes and Preliminary Findings

**DOI:** 10.3390/d15030328

**Published:** 2023-02-23

**Authors:** Magdalena Spießberger, Stephan Burgstaller, Marion Mesnil, Michael S. Painter, Lukas Landler

**Affiliations:** 1 Institute of Zoology, University of Natural Resources and Life Sciences (BOKU), 1180 Vienna, Austria; 2 Department of Game Management and Wildlife Biology, Faculty of Forestry and Wood Sciences, Czech University of Life Sciences Prague, Kamýcká 129, 165 00 Prague, Czech Republic; 3 UniLaSalle, Polytechnical Institute, Campus of Beauvais, 19 Rue Pierre Waguet, 60000 Beauvais, France; 4 Department of Biology, Barry University, 11300 NE 2nd Ave, Miami, FL 33161, USA

**Keywords:** urban habitat, movement, home range, *Bufotes viridis*, Bufonidae, circadian activity

## Abstract

Advancements in tracking technologies provide an increasingly important tool in animal monitoring and conservation that can describe animal spatial behavior in native habitats and uncover migratory routes that otherwise may be difficult or impossible to map. In addition, high-resolution accelerometer sensors provide powerful insights into animal activity patterns and can help to identify specific behaviors from accelerometer profiles alone. Previously, such accelerometers were restricted to larger animals due to size and mass constraints. However, recent advances make it possible to use such devices on smaller animals such as the European green toad (*Bufotes viridis*), the focus of our current study. We deploy custom made tracking devices, that consist of very-high-frequency transmitters and tri-axial accelerometers, to track toads in their native urban environment in Vienna (Austria). A total of nine toads were tracked, ranging from three to nine tracking days per individual during the post-breeding season period. We demonstrate that our devices could reliably monitor toad movement and activity during the observation period. Hence, we confirmed the predominantly nocturnal activity patterns and recorded low overall movement at this urban site. Accelerometer data revealed that toads exhibited brief but intense activity bursts between 10 pm and midnight, resting periods during the night and intermittent activity during the day. Positional tracking alone would have missed the major activity events as they rarely resulted in large positional displacements. This underscores the importance of and value in integrating multiple tracking sensors for studies of movement ecology. Our approach could be adapted for other amphibians or other animals with mass constraints and may become standard monitoring equipment in the near future.

## 1 Introduction

An important aspect of the life history of many animals are reoccurring movement and migration events [1–3]. For example, animal spatial behavior is known to play an important role in the distribution of individuals, influence resource allocation, affect population connectivity and, therefore, gene flow between populations [4]. Hence, disruption of animal movement corridors can lead to the isolation of populations, that can cause inbreeding depression [5,6]. Furthermore, reduced genetic variability in such isolated populations may reduce resilience and could eventually facilitate population extinction [7,8].

For many amphibians, including anurans, seasonal migrations are an essential, yet complex, component of their life history. For example, in temperate regions many anuran species migrate from their overwintering grounds to intermediate breeding sites, then continue their journey to summer habitats [9]. In anthropogenically altered areas, resulting in non-continuous and fragmented environments, amphibian migration and movement is often restricted to small patches of suitable habitats [10,11]. Emigration from such suitable patches may easily lead to the death of the individual, which might thereby select against more mobile phenotypes. Therefore, one could hypothesize that urban and rural populations of the same amphibian species might exhibit different spatial behavior, with reduced movement and activity in the former compared to the latter habitat. Only a few amphibian species, including the European green toad (*Bufotes viridis*), the focus of the current study, are known to inhabit both, core urban environments and their natural rural habitats. Green toads can be found in several cities across Europe [12–14], while their natural habitats are mainly steppes and wild river systems [15]. In their natural habitats, green toads are known for their highly mobile behavior and large home ranges [15–17]. In cities, they tend to suddenly appear at sites where no recent presence was reported [18] and may be frequent visitors to cleared and/or disturbed areas, such as construction sites [19]. The aim of the current study is to develop and deploy a miniaturized tracking system designed specifically for amphibians and to collect movement data from free-roaming toads inhabiting urban areas. This preliminary work will provide the foundation for future studies of amphibian movement and tracking that will likely shed light on their population dynamics and help to shape conservation efforts.

In general, there are several validated methods available to investigate animal movement. These include capture-mark-recapture (CMR) analyses and remote tracking (GPS, RFID and very-high-frequency (VHF) telemetry). While CMR can identify animal movement between known habitats and dispersal [20,21] as well as aiding in estimating home range size [22], remote tracking techniques can provide a more detailed characterization of the movement paths and unveil previously unknown habitat preferences or refuges of the target species during movement [23–26]. Furthermore, integrating tri-axial accelerometers into animal tracking systems can help to estimate energy expenditure, quantify levels of activity across various temporal scales and identify or distinguish between diverse behaviors [27–29]. Although the technology underlying tri-axial accelerometer sensors is no longer considered novel, reductions in size and mass, increased recording resolution and precision and methods of interpreting large volumes of data stored on accelerometer devices have made them a valuable tool for behavioral research. For example, accelerometers have been used to answer fundamental questions regarding energetics and behavioral strategies in apex predators [30,31]. In cane toads (*Rhinella marina*), it has been shown that the vector sum of acceleration (i.e., vectorial dynamic body acceleration, VeDBA) is highly positively correlated with energy expenditure, measured as oxygen consumption [32]. Furthermore, advancements in data processing and machine learning techniques have allowed researchers to use accelerometer data to identify ecologically relevant behaviors without the need for direct observation [33–35]. When accelerometer data is time-synced and combined with other streams of sensor data, important and sometimes surprising discoveries can be made, e.g., frigatebirds sleep mid-flight [36]. The application of these techniques in field studies provide a wealth of in-depth spatial, behavioral and physiological insight into animal ecology, and will be particularly valuable in studies involving smaller species, for which this technology is just now scaling down to suitable size and mass (e.g., cane toads’ mass is usually >1kg).

We designed a custom-made, ultra-light tracking system that integrated VHF transmitters and tri-axial accelerometers to monitor and track green toads in a core urban habitat over several days. Our approach made it possible to gain in-depth insights into the behavioral ecology of amphibians, inaccessible using previous methodologies, and we hope this preliminary work will catalyze the implementation of these tracking and analysis techniques for even smaller and/or more cryptic animals in future studies.

## 2 Materials and Methods

### 2.1 Study Species

**Figure 1:**
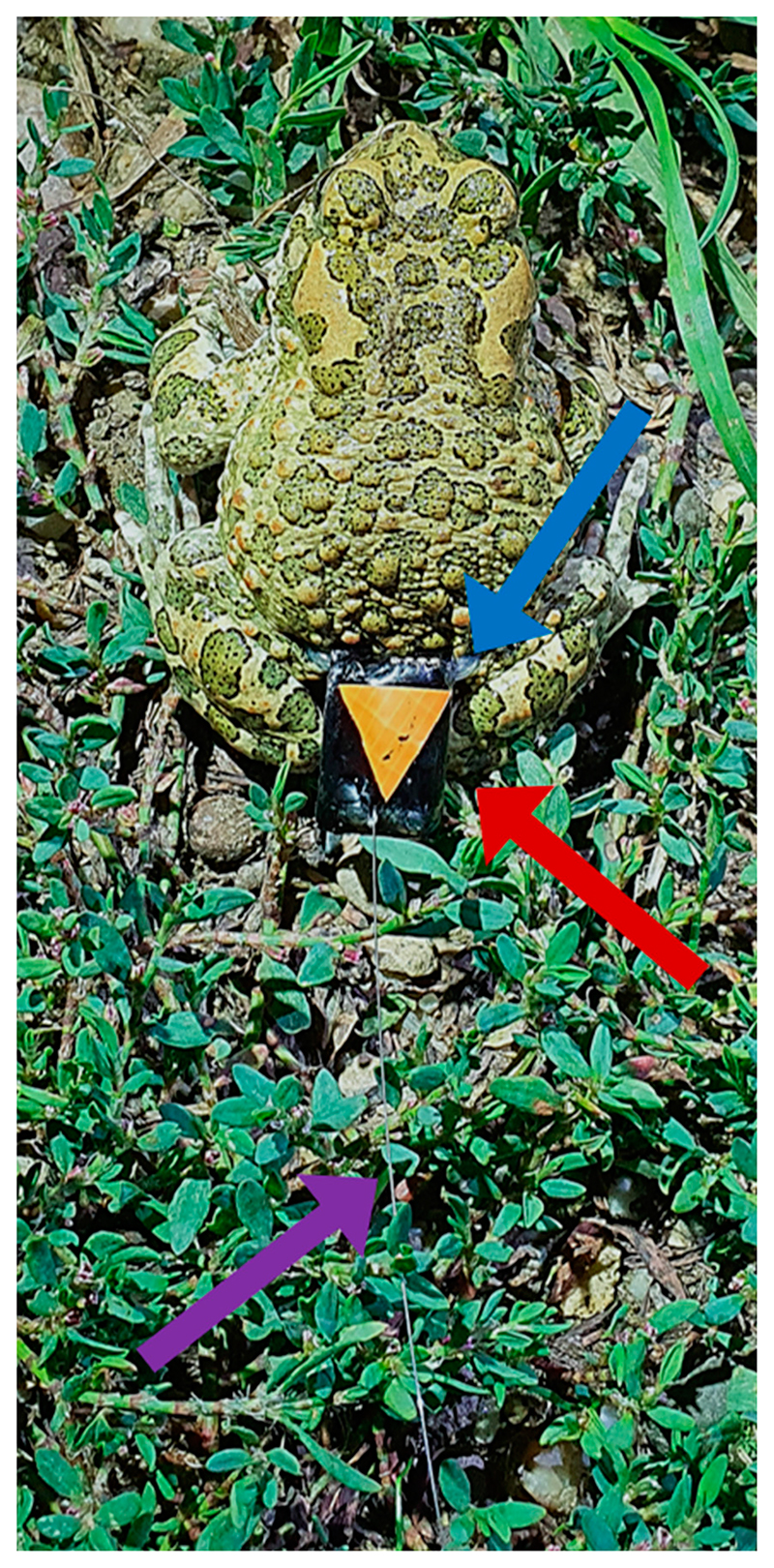
The tracking device attached to toad number K1 (male, mass: 35 g, snout-vent-length: 73 mm). The blue arrow points towards the silicon tubing (fastened using pre-made grommets) used to secure the tracking devices to the animal. The red arrow shows the tracking device and the purple arrow indicates the antenna (maximum length: 15.5 cm). Labels, in this case a yellow triangle, were used on each device to reliably distinguish between individual tracking units.

The green toad (*B. viridis viridis*, see Figure 1) is listed in appendix IV of the habitat directive of the European Union and is locally endangered in Europe [37]. However, counterintuitively to this classification status, they are frequently observed in anthropogenically disturbed areas, such as construction sites and quarries [19,38,39]. In Vienna, they occur close (~2.6 km) to the city center and thus several monitoring and conservation efforts are ongoing to preserve these urban populations [18,40]. Green toads are described as a predominantly nocturnal species with a primary breeding season between April and July [15]. They are a pioneer species capable of inhabiting dynamic environments with low predator prevalence [41,42]. Green toads show an ability to cope with unfavorable water chemistry (high salt concentrations), enabling them to inhabit the entire gradient of anthropogenic disturbance from pristine to urban environments [16,18,43].

### 2.2 Study Site

**Figure 2:**
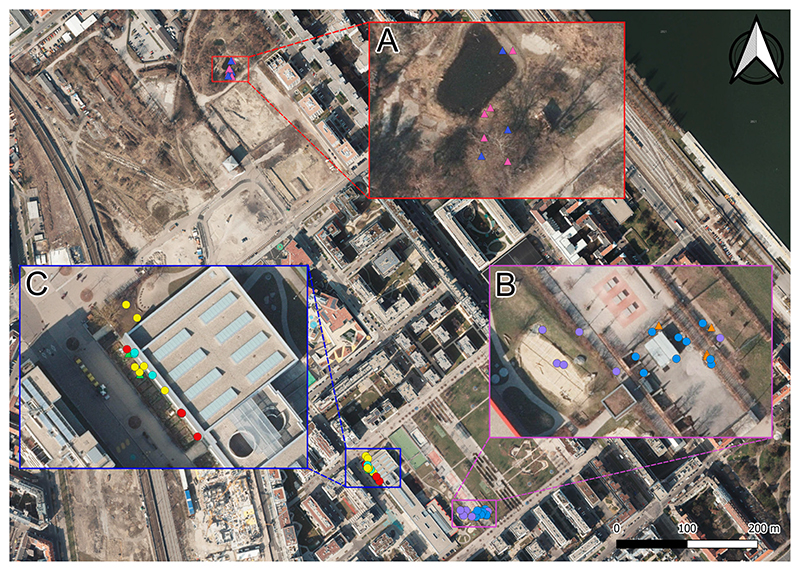
The study area and individual toad locations. The inset (**A**) is a zoomed in view of the Freie Mitte area with the animals K4 (pink rectangle, male) and K1 (blue rectangle, male), inset (**B**) shows the main area close to the spawning area with the animals K3 (orange rectangle, male), K5 (blue dot, male) and K8 (purple dot, female) and inset (**C**) the area behind the school in some small gardens with the animals K2 (green rectangle, male), K6 (red dot, male), K7 (yellow dot, female) and K9 (cyan dot, female). Map taken from [48].

Our study site was in the second district of Vienna (Austria) in the Rudolf Bednar Park (RBP), as well as two adjacent breeding sites (48.226° N, 16.397° E; Figure 2). The Rudolf Bednar Park is an intensely managed urban park. It has an area of approximately 3 ha and features shallow ornamental ponds, several hedges, flowers and communal gardens. Most of the area is covered by short-trimmed lawn. The adjacent breeding sites consist of two ponds at about 500 m from RBP (Figure 2). They are surrounded by an area with strong fallow/wasteland characteristics.

### 2.3 Tracking Device

We used a combination of an accelerometer, a magnetometer (Axy 5; Technosmart Europe srl, Rome, Italy) and a VHF transmitter (200 μW; Plecotus Solutions GmbH, Freiburg, Germany, exact frequencies used ranged from 149.997 to 150.199) connected to one rechargeable 20 mAh lithium-ion battery (designed and assembled by Plecotus Solutions GmbH, size of entire device: length: 18 mm, width: 12 mm and height: 10 mm). The VHF transmitter included a titanium antenna extending from the posterior end of the body that ranged in length from 12.5 to 15.5 cm (Figure 1). Antenna lengths of 15 cm would optimize the efficiency of the 150 MHz signal transmitted by each tracking device, however, minor adjustments to each unit were necessary to prevent the antennas from contacting the ground, resulting in the small variation in antenna length. Magnetometer data, although recorded, were not analyzed in the current study. Fully charged, the unit’s lifetime was approximately 12 days when programmed to our data sample rate (10 Hz accelerometer, 2 Hz magnetometer, ±8 * 9.81 ms^–2^, 10 bit) and recording continuously (every 0.1 s). Accelerometer data were stored on the device and retrieved via USB connection. Field trials were intentionally ended, at most, nine days after deployment. The tracking device was mounted on the toads around the waist using silicon tubing (diameter 2 mm, Figure 1). Silicon tubing is a common material to mount tracking devices on amphibians and appears very tolerable by amphibian skin [44]. In initial attempts prior to this study, we had already successfully tested the material and observed no damage of the toads’ skin or any other health issues. Animals were observed for at least 10 min after attaching the device and during this monitoring period all toads recovered from handling and device mounting quickly and resumed normal behavior within 10 min. Furthermore, toads had no skin irritations or similar signs of skin damage when the devices were recovered and removed. The total mass including silicone tubing was below 3 g (range: 2.54 to 2.92) for all units deployed in this study. We aimed for a relative unit mass less than 10% of the toad body mass. While lower percentages have been suggested for other animal groups [45], this is a commonly used value for amphibian tracking [44]. It allows observation of natural behavior without harming the animals and has been confirmed as suitable for our study species [46].

### 2.4 Field Methods

The field work took place after the green toads’ main breeding season, between 1 and 6 of August and the 15 and 23 of August 2022. The sunset time (given in UTC+2, according to local daylight savings time) for this period ranged from 8:31 pm (1 August) to 7:54 pm (23 August), while sunrise time ranged from 5:30 am to 6:00 am (taken from www.zamg.ac.at accessed on 12 January 2023). In total, 9 toads (3 females, 6 males) were tracked for a total of 15 days. Toad locations were identified using VHF homing-in using a handheld Yagi antenna (Lotek, 148–152 MHz) together with an RX98 receiver (TVP Positioning AB, Lindesberg, Sweden), and ultimately confirmed by visual detection. This was done once during daytime (between midday and 6:30 pm) to determine their refuges and several (one to four) times after sundown (between sundown and midnight). Toads were collected and tracking devices were immediately secured to individuals between 9:53 p.m. and 11:50 p.m. The animals were named consecutively K1 to K9 with K7, K8 and K9 being the 3 females in the study. K1 and K4 were collected in aquatic habitats and the others in their terrestrial habitat. The device on K2 unexpectedly stopped recording after 23 min and was recovered only by coincidence. As such, this individual was not included in the analysis. We photographed, weighed and measured the length of all individuals before the devices were mounted on the animals (start of tracking period) and again just before the devices were removed (end of tracking period). We used these data to calculate the green toad specific scaled mass index (a measure of body condition) as in [47]. One outlier data point (most likely a lapsus calami in the field notes) was removed.

### 2.5 Tracking Analysis

All location data were fed into QGIS (3.22.4) and plotted on Geoland Basemap Orthofoto (resolution: 1:5600.8) [48]. We processed the accelerometer data using the software X Manager (Technosmart Europe, Italy), and converted it for further analysis into the DDMT file format and extracted data using the software Daily Diary Multiple Trace (DDMT) (Wildbyte Technologies, Swansea, UK). All further processing of the data was performed in R [49]. The packages tidyverse [50] and data.table [51] were used for data preparations, lubridate [52] was used for handling dates and times and plotting was performed using ggpubr [53], patchwork [54] and scales [55]. For activity analysis, VeDBA (Vectoral Dynamic Body Acceleration) values were first time averaged in 10-min bins, then the mean (±SD) VeDBA for the 24-h periods for each individual was calculated (e.g., an animal tracked over five days would have a total of five mean VeDBA, each of which were averaged across 10-min bins per day). This was done to better visualize animal activity over the course of a 24 h circadian cycle.

The spatial activity range (i.e., “home range”) was evaluated using the package adehabitatHR [56]. the GPS coordinates were first transformed to the Austrian lambert projection and the 95% minimum convex polygon (MCP) in m^2^ and then calculated using the function mcp. Further, the range, mean and SD of the MCPs of all animals tracked were calculated. The MCPs are later referred to as ‘activity range’, which should be viewed with caution due to the intentionally short tracking period. The true home range is likely larger than the activity range found in our study and could be better estimated over longer tracking periods that include multiple seasons.

## 3 Results

The average toad mass and snout-vent-length of all animals included in the analysis was 39.64 g (SD: 8.21 g) and 73.13 mm (SD: 5.99 mm), respectively. Tracking devices, including silicon tubing, weighed on average 7.3% (SD: 1.4%) of toad mass with a range between 4.8% and 8.9%. The tracking devices did not negatively affect the green toads’ body condition, i.e., SMIs before (mean: 21.07 (SD: 2.50)) and after (mean: 21.85 (SD: 2.78)) the experiments were comparable (SMI change: + 0.78 (SD: 0.90)).

The calculated activity ranges (MCPs) for the tracking data were 74.36 m^2^ (SD: 35.30 m^2^, n = 8) with a range between 0 to 93.92 m^2^. Hence, the movement area during the time of observations was restricted to the immediate surrounding (Figure 2).

**Figure 3:**
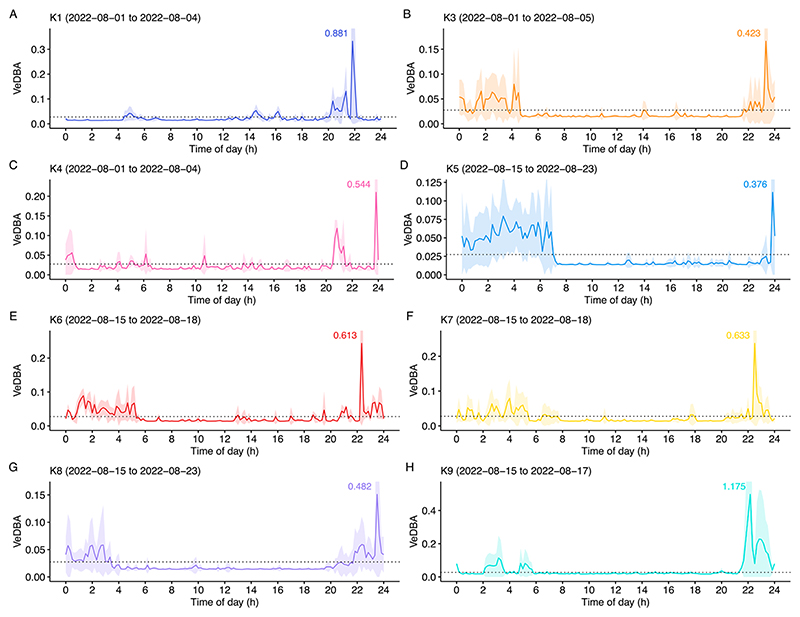
Average daily VeDBA for each individual (K1, K3 to K9 with the labels (**A**–**H**), respectively). Toad ID and tracking date range is shown on the top left in each panel. Graph color corresponds to colors in Figure 2. To improve visual representation, the upper ranges of SDs are cut-off. However, the maximum SD for each graph is provided next to the peaks. Note that the y-axis scales differ between individuals. The solid-colored line represents the mean VeDBA and the shaded area represents the standard deviation (SD). The horizontal dotted lines in each plot represent the overall mean VeDBA, calculated across all eight toads in the study, which was 0.027. K1 and K3 to K6 were males. K7 to K9 females.

However, we observed accelerometer traces consistent with expectations for a primarily nocturnal species that showed an onset of activity shortly after 8 pm and a decrease in activity around 7 am at the latest (Figure 3). Several individuals showed intermittent resting periods during their main activity phase, either at the start or the end of the night, as well as minor activity periods during the day, often in the afternoon hours. The highest activity peak by far occurred between 10 pm and midnight (around two and four hours after sunset). This was true for all animals in this study. However, there was a high degree of variation in absolute maximum VeDBA values between individuals (Figure 3).

## 4 Discussion

Our results show that accelerometer-assisted tracking can be conducted in small vertebrates, i.e., anurans, over the course of at least several days. In general, green toads are known as primarily nocturnal animals [15], which was confirmed in our study by the accelerometer profiles. Exceptions from nocturnal activity are examples of calling behavior during the main breeding season (LL personal observation and see reported observation in [15]), a period not included in the current study. Within the sample dates used in the current study, the main activity periods took place during the night. However, this activity was disrupted by intermittent resting periods. Notably, we also observed phases of activity during the day for each of the individuals. We observed the highest peak of activity (possibly including movements without positional change, e.g., turning, as well as movements between locations) between 10 pm and midnight (corresponding to about two to four hours after sunset). Such peaks were short (less than one hour) and were not repeated during the remainder of the 24 h, although some activity was recorded at much lower intensities throughout the day. Interestingly, Hemmer and Kadel [57] reported, from terraria observations after the breeding season, two activity peaks, one shortly after 8 pm and another around 10 pm, with slowly increasing activity from morning towards sundown. In contrast, Jungfer [58] reported three main activity phases for the green toad in laboratory experiments, corresponding to morning, midday and evening. In his experiments this was similar to activity patterns of the common toad (*Bufo bufo*), while the natterjack toad (*Epidalea calamita*) only showed one broad activity maximum in the morning and one minor activity peak during the night. However, the main activity phases during the day for all three species monitored in this study stand in stark contrast to the general nocturnal activity reported in several other studies summarized in [15,59,60]. Hence it is plausible, and suggested by Hemmer and Kadel [57], that the activity patterns reported in Jungfer [58] were caused by other factor(s), e.g., humidity, temperature, feeding regime, disturbances. While the methodology developed and tested in the current study provides several advantages over laboratory studies, including the high sampling frequency of activity and observing behavior in natural habitats under natural contexts, there are also possible limitations, especially when using our tracking devices for other species. The current size and mass of the device might exceed the maximum limits for several amphibian species and could influence behavior, e.g., reduce activity due to the extra load or affect foraging and/or reproductive behavior. The relatively short battery life could limit the scale and scope of certain studies, as we had to recollect the devices within a week, approximately, to recharge the batteries. Furthermore, the accelerometer data is stored on the device itself, and therefore, the trackers need to be manually retrieved to obtain the data [61]. Finally, similar to other VHF transmitters, buildings, dense vegetation, and water can interfere with the transmitter signals and reduce the accuracy or reliability of individual detections, e.g., [62].

Green toads were mainly stationary in our study, presumably because the field work took place after the main breeding season. Moreover, weather conditions during this time were exceptionally hot and dry with little rainfall (2 mm) and temperatures up to 36 °C (min: 20.4 °C mean: 28.4 °C) in Vienna (taken from https://meteostat.net/ and https://www.accuweather.com, accessed on 12 January and 2 February 2023, respectively). The stationary behavior is also evident from the small activity ranges of the animals. Green toads are known as highly mobile species with home ranges as large as 1074 m^2^ to 3339 m^2^ in tracking studies (over 13.5 to 71 days per individual) using similar sized tags. These are about 15 to 50 times of the ranges we observed [16]. Hence, even for a post-breeding behavior and our short tracking period, this appears to be exceptionally low movement activity. It should be noted that distance of the land habitats to the next breeding sites in the study area are very short (between 12 and 120 m). Therefore, these toads would not have to migrate far to participate in the breeding activity in the next season. Hence, the activity range, even when including the breeding migration, can be assumed to be extraordinarily small.

The lack of movement could be an adaptation to the urban environment to avoid mortality by roadkill, one of the major threats to amphibian wildlife worldwide [63–65]. Furthermore, an overall decrease in activity could also lower energy expenditure, which might be advantageous for toads in urban environments, usually offering lower prey abundance [66]. A tendency to reduced activity range sizes in more urban environments has been demonstrated in other species, such as racoon, key deer and stonemarten [67–69]. The stationary and nocturnal, and therefore ‘hidden’, post-breeding lifestyle of the observed green toads is consistent with this possibility. The sudden occurrences of green toads at urban sites [18] could therefore, at least partially, be based on a low density of hidden green toads throughout the city area. Such a presence could be a legacy effect of former suitable habitats in the area, e.g., wild river systems and/or open land and steppes. However, at this stage, this is speculative and would need to be further investigated, for instance by comparisons of accelerometer data of toads in urban areas and more natural habitats.

We observed individual differences with respect to the magnitude of activity, though the overall patterns were comparable. The reasons for this are unclear in this preliminary study and might average out with data over a longer period. However, such differences could correspond to different temporary behavioral states or even different behavioral syndromes, which are known from amphibian spatial behaviors [70–72]. However, more individual data over a longer period would be needed to confirm consistent individual differences.

In conclusion, our study exemplifies the use of accelerometer-assisted tracking in a small vertebrate. The detailed activity records made possible by these advancements in technologies increase our insight into behavior, especially the intermittent activities during the day would have been missed by positional tracking alone. Accelerometers are a promising tracking tool to investigate animal behavior in the wild and can provide in-depth insights into the activity patterns and potentially energy expenditure of animals in their native habitats. Furthermore, when coupled with additional sensors (e.g., VHF transmitters, magnetometers), researchers can obtain a wealth of spatial and behavioral data from animals in natural contexts. Further technical developments might expand the possible range of animals that can be used and make it a standard tool in animal movement studies.

## Supplementary Material

Positional data

VeDBA data

## Data Availability

Data are available in the main manuscript and Supplementary information.
